# Prenatal alcohol and tetrahydrocannabinol exposure: Effects on spatial and working memory

**DOI:** 10.3389/fnins.2023.1192786

**Published:** 2023-06-13

**Authors:** Annie Lei, Kristen R. Breit, Jennifer D. Thomas

**Affiliations:** ^1^Department of Psychology, Center for Behavioral Teratology, San Diego State University, San Diego, CA, United States; ^2^Department of Psychology, West Chester University of Pennsylvania, West Chester, PA, United States

**Keywords:** cannabis, alcohol, combined exposure, spatial memory, working memory, pregnancy, vaping, rodents

## Abstract

**Introduction:**

Alcohol and cannabis are widely used recreational drugs that can negatively impact fetal development, leading to cognitive impairments. However, these drugs may be used simultaneously and the effects of combined exposure during the prenatal period are not well understood. Thus, this study used an animal model to investigate the effects of prenatal exposure to ethanol (EtOH), Δ-9-tetrahydrocannabinol (THC), or the combination on spatial and working memory.

**Methods:**

Pregnant Sprague–Dawley rats were exposed to vaporized ethanol (EtOH; 68 ml/h), THC (100 mg/ml), the combination, or vehicle control during gestational days 5–20. Adolescent male and female offspring were evaluated using the Morris water maze task to assess spatial and working memory.

**Results:**

Prenatal THC exposure impaired spatial learning and memory in female offspring, whereas prenatal EtOH exposure impaired working memory. The combination of THC and EtOH did not exacerbate the effects of either EtOH or THC, although subjects exposed to the combination were less thigmotaxic, which might represent an increase in risk-taking behavior.

**Discussion:**

Our results highlight the differential effects of prenatal exposure to THC and EtOH on cognitive and emotional development, with substance- and sex-specific patterns. These findings highlight the potential harm of THC and EtOH on fetal development and support public health policies aimed at reducing cannabis and alcohol use during pregnancy.

## 1. Introduction

Prenatal alcohol exposure presents a substantial risk to the developing fetus, leading to a variety of adverse outcomes in physical, cognitive, and behavioral development collectively known as fetal alcohol spectrum disorders (FASD). Prevalence of FASD is alarmingly high, estimated at 2–5% in the US and several Western European countries (May et al., 2009, 2018). Despite the known risks, it is estimated that 11–14% of expectant individuals are currently drinking, with 5% engaging in binge drinking (Gosdin, 2022; Substance Abuse and Mental Health Services Administration, 2022); thus, FASD represents a serious and persistent public health problem. However, prenatal alcohol exposure does not always occur alone. In fact, cannabis is the most commonly used illicit substance during pregnancy (Alpár et al., 2016), with 8% of pregnant individuals reporting use within the past month (Substance Abuse and Mental Health Services Administration, 2022). Pregnant individuals who use cannabis typically perceive no risk, are early in their pregnancy, and frequently co-use with tobacco and/or alcohol (Odom et al., 2020). This frequent co-use with alcohol is of great concern.

Co-use of alcohol and cannabis may be reflected in two distinct patterns of substance use: SAM (simultaneous alcohol and marijuana use) and CAM (concurrent alcohol and marijuana use). SAM involves using both substances close in time so that their effects overlap, whereas CAM refers to using both substances, but not necessarily on the same occasions (Bravo et al., 2021; Gonçalves et al., 2022). Both patterns of use increase the vulnerability to negative consequences (Jackson et al., 2020; Bravo et al., 2021), but SAM, with almost twice the prevalence of CAM (Subbaraman and Kerr, 2015), is more deleterious and associated with more severe consequences than those related to CAM or alcohol-only use (Jackson et al., 2020; Bravo et al., 2021; Gonçalves et al., 2022; Salguero et al., 2022).

Simultaneous use of alcohol and cannabis is associated with increased frequency of use for both substances and an increase in the quantity of alcohol consumed (Subbaraman and Kerr, 2015; Crummy et al., 2020). Furthermore, individuals who engage in simultaneous use demonstrate increased negative consequences related to alcohol (i.e., physical altercations, legal issues, car accidents, risky sexual behavior, negative academic outcomes, hangovers) potentially due to their propensity for binge-drinking behavior (Salguero et al., 2022), while also reporting more subjective alcohol-related positive consequences (i.e., relaxation, sociability, feeling buzzed; Lee et al., 2022).

With the legalization of recreational cannabis, prevalence of simultaneous co-use has increased in adults (Gonçalves et al., 2022). Recent estimates indicate that 20% percent of females who are current non-heavy drinkers use both alcohol and cannabis, whereas almost 50% of female heavy drinkers consume both drugs (Substance Abuse and Mental Health Services Administration, 2022). The high levels of co-use of alcohol and cannabis is concerning, particularly given that approximately half of all pregnancies are unplanned (Finer and Zolna, 2016) which can result in unintended prenatal drug exposure. In fact, among pregnant individuals consuming cannabis, half also report consuming alcohol (Substance Abuse and Mental Health Services Administration, 2015). Notably, these data are based on self-reports and given the possibility that stigma and legal implications may influence responses, use rates of either alcohol or cannabis may be much higher (Sharma et al., 2016; Chiandetti et al., 2017; England, 2020).

Prenatal exposure to either alcohol or cannabis can influence a range of behavioral domains (Fontaine et al., 2016; Mattson et al., 2019; Little et al., 2021; De Genna et al., 2022). For example, cognitive dysfunction associated with prenatal alcohol exposure can contribute to life-long challenges in school and independent living (McLachlan et al., 2020), negatively affecting an individual’s quality of life (Mattson et al., 2019). Specifically, clinical studies have shown that prenatal alcohol exposure disrupts learning and memory on a variety of tasks, including spatial memory (Dodge et al., 2019) and spatial working memory (Moore et al., 2021). Prenatal alcohol exposure can lead to difficulties in efficiently encoding information (Lewis et al., 2021), which is associated with slower information processing and/or utilization of inefficient and ineffective memory strategies (Lewis et al., 2021). Similarly, preclinical research confirms that prenatal alcohol exposure leads to spatial learning (Schambra et al., 2017; Aglawe et al., 2021) and working memory deficits (Schambra et al., 2017; Waddell et al., 2020; Gursky et al., 2021), consistent with the vulnerability of the developing hippocampus, a brain region important for learning and memory (Fontaine et al., 2016; Mattson et al., 2019; Dodge et al., 2020), and the prefrontal cortex, a brain region important in executive functioning including working memory (Mattson et al., 2019; Waddell et al., 2020; Gursky et al., 2021).

The effects of prenatal cannabis exposure on memory and other cognitive functions are less understood. Although some clinical studies report only weak associations or a lack of clinically relevant impairments (Sharapova et al., 2018; Navarrete et al., 2020; Torres et al., 2020; Murnan et al., 2021; Betts et al., 2022; De Genna et al., 2022), others report that prenatal cannabis exposure is related to memory deficits (Sharapova et al., 2018; Grant et al., 2020; Navarrete et al., 2020; De Genna et al., 2022). Notably, one fMRI study found heightened neural activity among subjects prenatally exposed to cannabis when performing a working memory task, despite no group differences in task performance (Smith et al., 2016). This suggests a possible compensatory response for equivalent task completion compared to non-exposed individuals, although others have failed to replicate this finding (Cioffredi et al., 2022). Importantly, the adverse effects of cannabis use during pregnancy are most often seen with comorbid substance use (Basavarajappa, 2015; Forray, 2016), and so effects on memory may be more severe with combined exposure to alcohol.

Similar to clinical studies, preclinical studies also vary, with some studies finding no effects of prenatal cannabis (Breit et al., 2019b), whereas others finding persistent deficits in learning and memory which may be due to alterations in the prefrontal cortex and hippocampus (Beggiato et al., 2017, 2020; de Salas-Quiroga et al., 2020; Grant et al., 2020; De Genna et al., 2022). Variation in findings may be due to the type of cannabinoid used. For example, prenatal CBD exposure may actually improve spatial working memory (Wanner et al., 2021). Thus, the effects of prenatal cannabis on learning and memory are still unclear.

Moreover, the effects of prenatal exposure to the combination of alcohol and cannabis is also not well understood, although given that ethanol can interact with the endogenous cannabinoid system (ECS) (Basavarajappa, 2015), the likelihood that the two drugs interact is high. Prenatal exposure to either substance separately can disrupt development of the hippocampus and prefrontal cortex by interfering with the endocannabinoid system (Basavarajappa, 2015; Smith et al., 2016; Beggiato et al., 2020; Manduca et al., 2020). THC exposure may weaken the developing nervous system, possibly acting as a “first hit” to the ECS, making the nervous system more vulnerable to other drug insults, as suggested by the “double hit hypothesis” (Richardson et al., 2016; Little et al., 2021). In fact, preclinical studies have found interactive effects of prenatal alcohol and cannabis on physical birth defects (specifically malformations of the eyes, face, and brain), even at low doses of both drugs (Fish et al., 2019). Our lab found that developmental exposure to the cannabinoid receptor agonist CP-55,940, which mimics the effects of THC, in combination with ethanol led to higher mortality rates, more severe growth deficits, and more severe behavioral deficits than exposure to either substance alone (Breit et al., 2019a,b). Additionally, the combination of prenatal THC and prenatal EtOH leads to more severe open field hyperactivity, specifically among males (Breit et al., 2022). In contrast, the Maternal Health Practices and Child Development (MHPCD) longitudinal study found that either prenatal exposure to ethanol or cannabis was an independent predictor of school performance, but found no interaction between prenatal marijuana and prenatal alcohol exposure (Goldschmidt et al., 2004). However, the MHPCD project began before the rapid rise in THC levels, not representing the levels consumed today.

To model ethanol and cannabis simultaneous co-exposure, the present study used an animal model with THC exposure via electronic cigarettes (e-cigarettes) vapor. The use of e-cigarettes for cannabis consumption is becoming more prevalent, particularly as more states legalize cannabis (Borodovsky et al., 2016). In fact, pregnant women perceive e-cigarettes as safer alternatives to use during pregnancy than combustible cigarettes (Baeza-Loya et al., 2014; Kahr et al., 2015), which is concerning because vaporized cannabis may lead to higher THC concentrations and greater pharmacodynamic effects compared to the traditional route of smoking (Spindle et al., 2018). Pregnant dams were exposed to alcohol, THC, or the combination throughout gestation (gestational days [GD] 5–20, a period of development equivalent to the first and second trimester for humans; Dobbing and Sands, 1979; Patten et al., 2014), and both spatial and working memory were examined in the offspring using the Morris water maze.

## 2. Materials and methods

All procedures included in this study were approved by the San Diego State University Institutional Animal Care and Use Committee and are in accordance with the National Institute of Health Guide for Care and Use of Laboratory Animals.

### 2.1. Subjects

Breeding took place in the mating colony at the Center for Behavioral Teratology, San Diego State University, as previously described in detail (Breit et al., 2020). Briefly, adult Sprague–Dawley females were housed with adult Sprague–Dawley males overnight until a seminal plug, indicating mating, was found and designated GD 0. The SAM model used in this study was developed to model human consumption patterns (Hartman et al., 2015; Andrenyak et al., 2017), generating low to moderate THC exposure, with peak alcohol and THC levels overlapping in time. The model has been previously described in detail in Breit et al. (2020). Pregnant dams were randomly assigned to receive vaporized EtOH (68 mL/h; Sigma-Aldrich), THC via e-cigarette (100 mg/mL using SMOK V8 X-Baby Q2; NIDA Drug Supply Program), the combination of EtOH and THC, or Vehicle (propylene glycol; Sigma-Aldrich). Dams were exposed daily from gestational days (GD) 5–20 via a vapor inhalation system (La Jolla Alcohol Research Inc.) for 3 h, 30 min. The first 3 h were EtOH or air and the last 30-min session were THC or vehicle delivered in 6-s puffs every 5 min. This co-exposure paradigm produced maternal plasma levels of 150–200 mg/dL for alcohol and 20 ng/mL for THC (Breit et al., 2020) and did not significantly impact maternal food or water intake.

There were no effects of EtOH, THC, or the combination on gestation duration, number of offspring, birth weight, or male to female litter ratio (Breit et al., 2020). Following birth, litters (EtOH + THC: 10; EtOH + Vehicle: 12; Air + THC: 13; Air + Vehicle: 12) were randomly culled to 8 pups (4 males and 4 females whenever possible) on PD 2. Subjects were tattooed for identification purposes on PD 7, to keep experimenters blind to treatment condition. To control for possible litter effects, only one sex pair per litter was randomly assigned to the Morris water maze spatial learning and working memory tasks (EtOH + THC: 9 females, 10 males; EtOH + Vehicle: 12 females, 12 males; Air + THC: 12 females, 14 males; Air + Vehicle: 12 females, 12 males).

### 2.2. Behavioral testing

#### 2.2.1. Morris water maze visuospatial learning

The Morris water maze apparatus consisted of a large circular tank (178-cm diameter, 61-cm height) painted black and filled with 26°C water. A Plexiglas escape platform (4-in diameter) was placed randomly in 1 of 4 quadrants and the location remained constant for each subject throughout the trials. The escape platform was 4 cm under the surface of the water, hidden from view of the subjects. The room contained a number of extra-maze cues mounted on the room walls. A video tracking system interfaced with the computer running the Water2020 software (HVS Image) was placed above the tank to record data.

Subjects were tested for 4 trials/day with a 3–5 min intertrial interval (ITI) for 6 consecutive days during PD 40–45. At the start of each trial, each subject was placed in the water, facing the edge of the tank at 12 potential random starting locations. If the subject could not locate the platform within 60 s, the experimenter manually guided the subject to the platform. The rat remained on the platform for 10 s before being removed. Path length, latency, heading angle, swimming speed and thigmotaxis served as outcome measures. Heading angle is the difference between initial swimming direction and direction of the escape platform and serves as a measure of spatial accuracy as it reflects the subject’s ability to select the most direct and efficient path to the target. Thigmotaxis (swimming along the outer perimeter of the tank) serves as a measure of anxiety. On the 7th day (PD 46), subjects were tested on a probe test trial, where the platform was removed and subjects were allowed to swim for 60 s. Time spent and passes through the target quadrants, as well as the target area (3 times the diameter of the platform), served as a measure of spatial memory.

#### 2.2.2. Working memory

From PD 55–60, the same subjects were then tested on a working memory version of the Morris water maze task (D’Hooge and De Deyn, 2001; Vorhees and Williams, 2006; Schulteis et al., 2008; Schneider and Thomas, 2016). For this task, subjects were tested for 2 sessions per day, one in the morning and one in the afternoon (6 h apart). Each session consisted of 1 acquisition trial and 1 test trial. During the acquisition trial, the Plexiglas escape platform was placed randomly in 1 of 8 positions. Once the subject found the platform, where they remained for 20 s, they were removed for either a 0-s ITI (first 3 days) or a 60-s ITI (last 3 days), before being placed back into the tank for the test trial. If subjects failed to find the platform with 60 s, they were guided to the platform. During each session, the location of the platform was moved to a novel location, placing a demand on working memory. Path length, latency, heading angle, swimming speed and thigmotaxis served as outcome measures.

### 2.3. Data analyses

Data were analyzed using SPSS software (SPSS Inc., Chicago, IL) with a significance value set at *p* < 0.05. All data were analyzed using 3-way (EtOH, Air) × (THC, Vehicle) × (Female, Male) ANOVAs. When applicable, data were also analyzed by Group (EtOH + THC, EtOH + Vehicle, Air + THC, Air + Vehicle), with Fisher’s Least Significant Difference *post hoc* tests as follow-up, given complex higher-order interactions. All working memory data were initially analyzed separately for 0-s ITI and 60-s ITI training and testing trials using 3 (Day) × 2 (Sex) × 2 (EtOH) × 2 (THC) ANOVAs. Day, Session, and Trial were used as repeated measures. Data were also analyzed separated by sex due to previous findings on sex differences (Breit et al., 2022). Means and standard errors of the mean (M ± SEM) are reported for data described but not shown graphically. Power analyses were conducted with power 0.80. Effect sizes for both the Morris Water Maze visuospatial learning data and for the working memory version of the task ranged from moderate to large (0.05–0.2).

## 3. Results

### 3.1. Body weight

**Figure 1:**
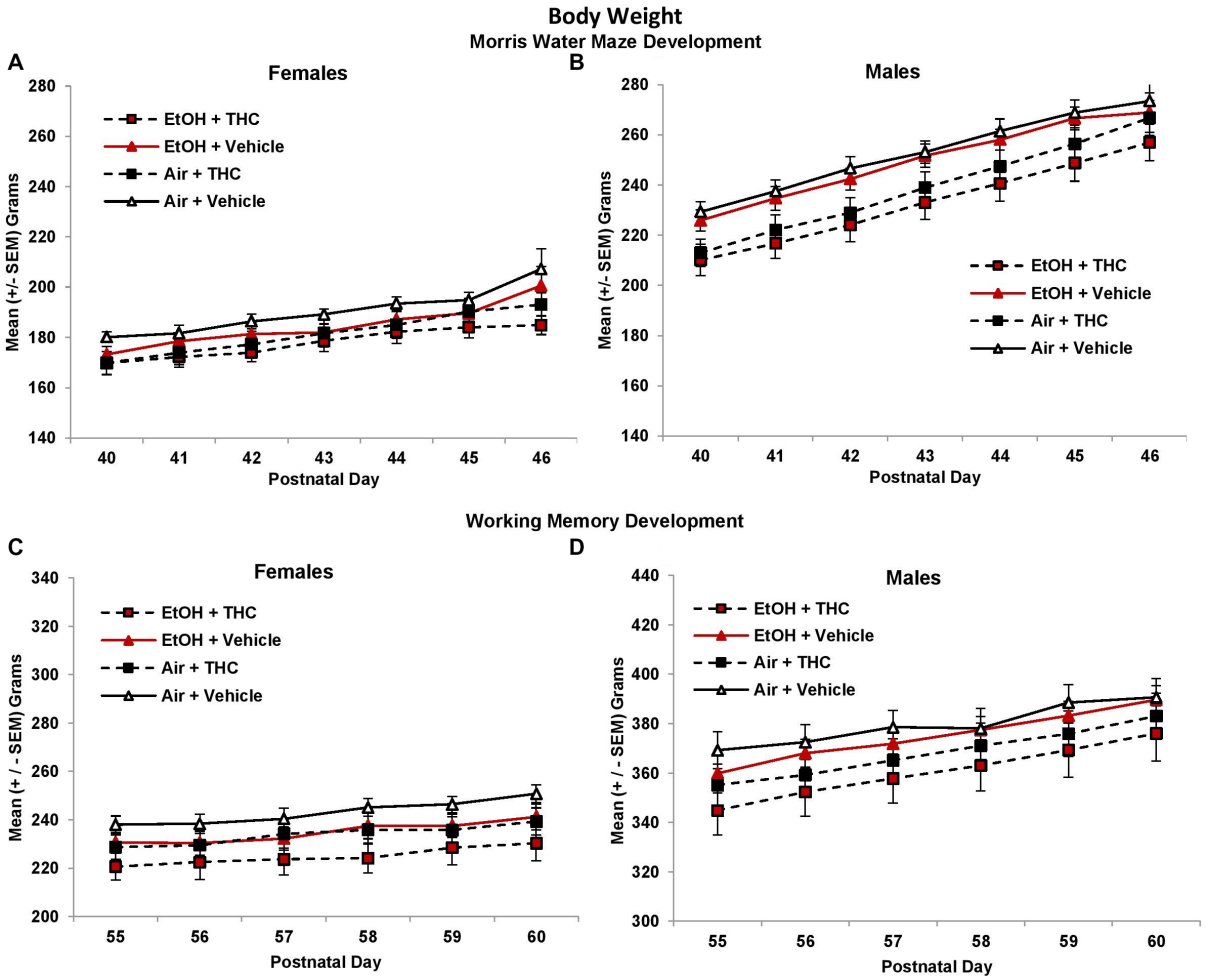
Offspring exposed to prenatal THC weighed less than those not exposed to prenatal THC throughout behavioral testing, although these effects became less pronounced with age [females **(A,C)**; males **(B,D)**].

During the Morris water maze testing, all groups gained weight across Days (*F*[6,510] = 293.4, *p* < 0.01). Although offspring exposed to prenatal THC weighed less than those not exposed to THC on each day (*F*[1,85] = 11.0, *p* < 0.01; Figure 1), there were interactions of Day*Sex*THC (*F*[6,510] = 4.0, *p* < 0.01) and Day*Sex (F[6,510] = 40.4, *p* < 0.01), as body weight deficits became less severe among the THC groups over days, particularly among males. In addition, females weighed less than males (*F*[1,85] = 296.1, *p* < 0.01) on each day. This pattern continued during the working memory testing {PD55-60; THC (*F*[1,85] = 5.3, *p* < 0.05); Sex (F[1,85] = 77.0, *p* < 0.01); Day*Sex*THC (F[5,425] = 2.4, *p* < 0.05); Day*Sex (F[5,425] = 35.5, *p* < 0.01)}.

### 3.2. Morris water maze

#### 3.2.1. Acquisition

**Figure 2:**
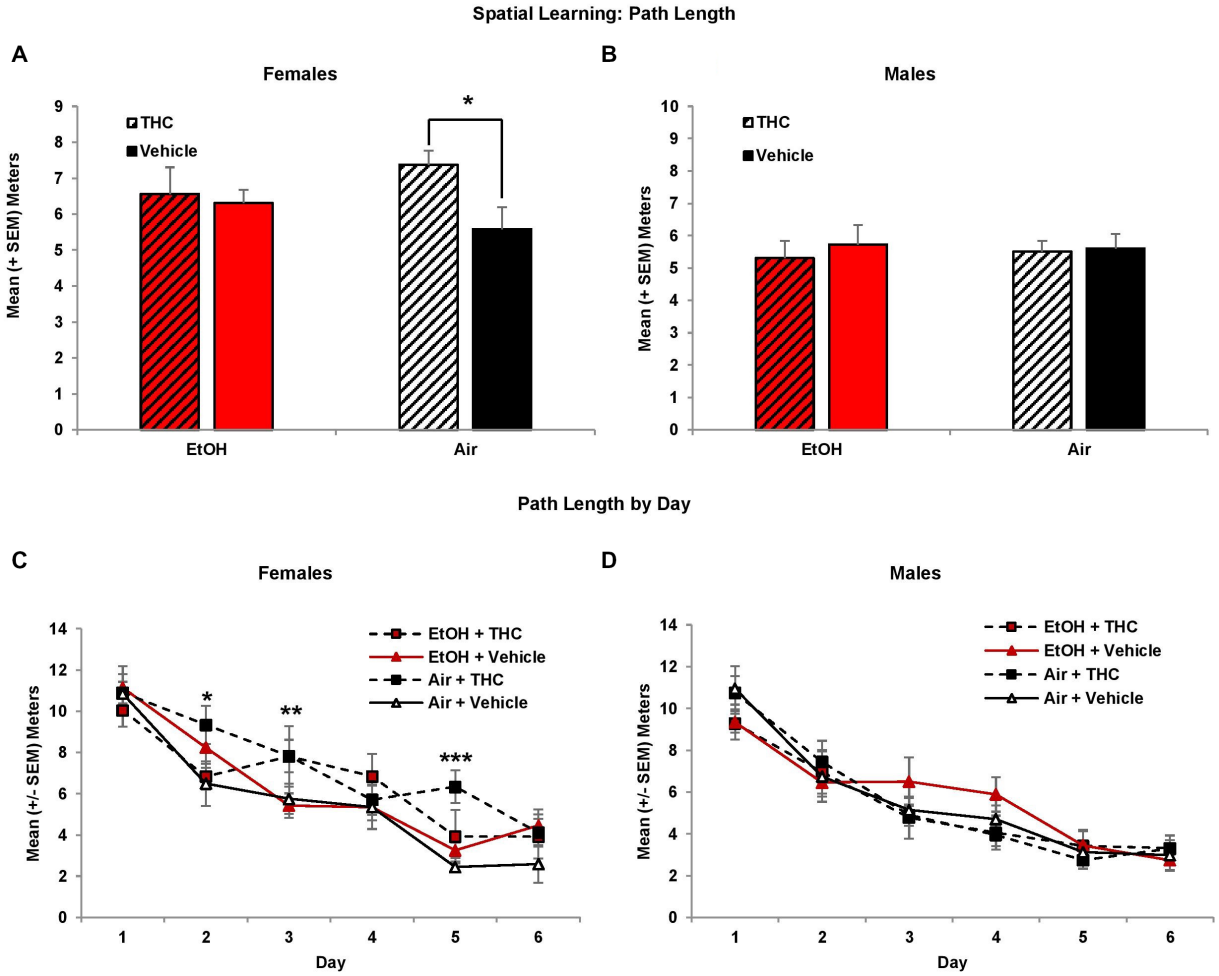
Female offspring prenatally exposed to THC alone required longer path lengths to find the hidden platform during the visuospatial memory task **(A,C)**. There were no effects of prenatal exposure on performance among male offspring **(B,D)**. *Air + THC > Air + Vehicle, *p* < 0.05; **Air + THC > EtOH + Vehicle, *p* < 0.05; ***Air+THC > all other groups, *p* < 0.01.

Although performance improved in all groups across acquisition days (*F*[5,425] = 106.9, *p* < 0.01), female subjects exposed to prenatal THC alone showed spatial learning deficits. There were no group differences in path length to find the platform on the first day of testing; however, THC-exposed females exhibited slower acquisition than non-THC exposed females, taking significantly longer paths to find the platform (Figure 2C). This produced an interaction of THC*Sex*Day (*F*[5,425] = 2.3, *p* < 0.05), and a main effect of Sex (*F*[1,85] = 6.6, *p* = 0.01). Although the interaction of EtOH and THC did not reach statistical significance, these effects were most robust within the THC only group. Females exposed only to prenatal THC took significantly longer path lengths compared to controls (*p* < 0.05; Figure 2A), whereas the combination group did not differ significantly from any other group. There were no significant group differences in performance among males (Figures 2B,D).

Similar findings were observed in the latency to find the hidden platform (data not shown), despite differences in swimming speed. Prenatal THC exposure tended to reduce swimming speed among the males (F[1,44] = 3.6, *p* = 0.07; EtOH + THC: *M* = 0.18 ± 0.01 m/s; EtOH: *M* = 0.20 ± 0.01 m/s; THC: *M* = 0.19 ± 0.01 m/s; Air + Vehicle: *M* = 0.20 ± 0.01 m/s), but not females. There was also a main effect of Sex (*F*[1,85] = 5.3, *p* < 0.05).

**Figure 3:**
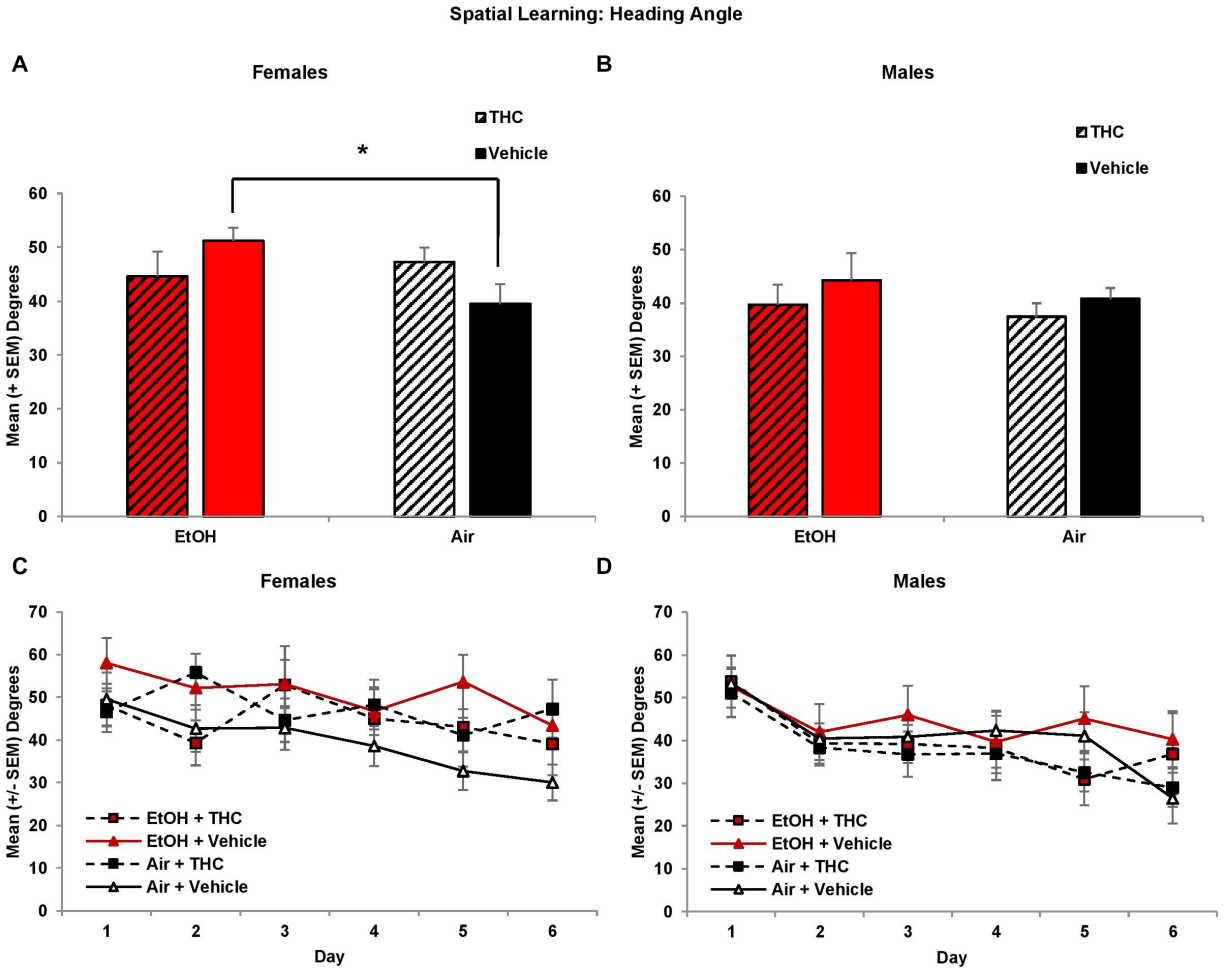
Female offspring prenatally exposed to alcohol alone had significantly larger heading angles (impaired performance) compared to controls **(A,C)**. Heading angle did not differ among groups of male offspring **(B,D)**. *EtOH+Vehicle > Air+Vehicle, *p* < 0.05.

Interestingly, there were main effects of Sex (*F*[1,85] = 4.5, *p* < 0.05), Day (*F*[5,425] = 8.0, *p* < 0.01) and Trial (*F*[3,255] = 8.3, *p* < 0.01) on heading angle. Given the main effect of Sex, analyses were conducted for each sex separately. Among females, the EtOH- only exposed group displayed significantly larger heading angles compared to controls, producing an EtOH*THC interaction (*F*[1,41] = 4.7, *p* < 0.05), as seen in Figures 3A,C. Heading angle was not significantly affected by prenatal THC or EtOH in males (Figures 3B,D).

**Figure 4:**
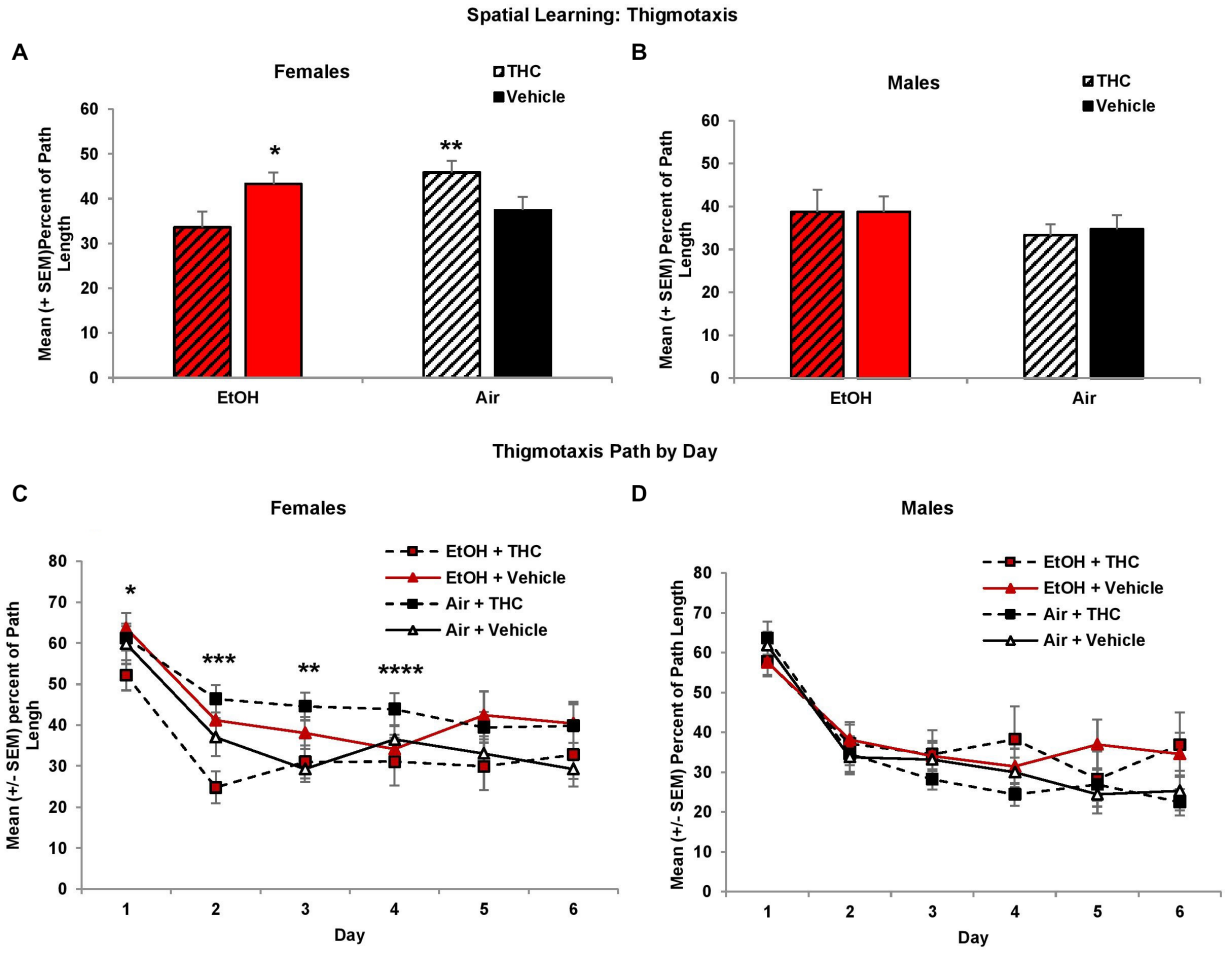
Female offspring exposed to either THC alone or alcohol alone were more thigmotaxic compared to those exposed to the combination or controls **(A,C)**. No differences were observed among male offspring **(B,D)**. *EtOH + Vehicle > EtOH + THC, *p* < 0.05; **Air + THC > EtOH + THC and Air + Vehicle, *p*’s < 0.05; ***all other groups > EtOH + THC, *p*’s < 0.05; ****Air + THC > EtOH+THC, *p*’s < 0.05.

Larger heading angle could indicate impaired spatial accuracy but could also be affected by differences in thigmotaxis (swimming along the outer perimeter of the tank due to anxiety). Interestingly, prenatal exposure to either THC or EtOH increased thigmotaxis among female offspring, leading to significant interactions of Sex*EtOH*THC (*F*[1,85] = 4.5, *p* < 0.05) and EtOH*Day (*F*[5,425] = 2.6, *p* < 0.05). Exposure to either THC or EtOH alone produced more thigmotaxis compared to the combination for the first 4 days of acquisition in females (Figure 4C). When collapsed across days (EtOH*THC, *F*[1,41] = 9.9, *p* < 0.01), females exposed to THC only were more thigmotaxic compared to controls and the combination group, whereas the EtOH group was more thigmotaxic than the combination group (*p*’s < 0.05), as seen in Figure 4A. These effects on thigmotaxis were not seen in the male subjects (Figures 4B,D).

#### 3.2.2. Spatial memory

**Figure 5:**
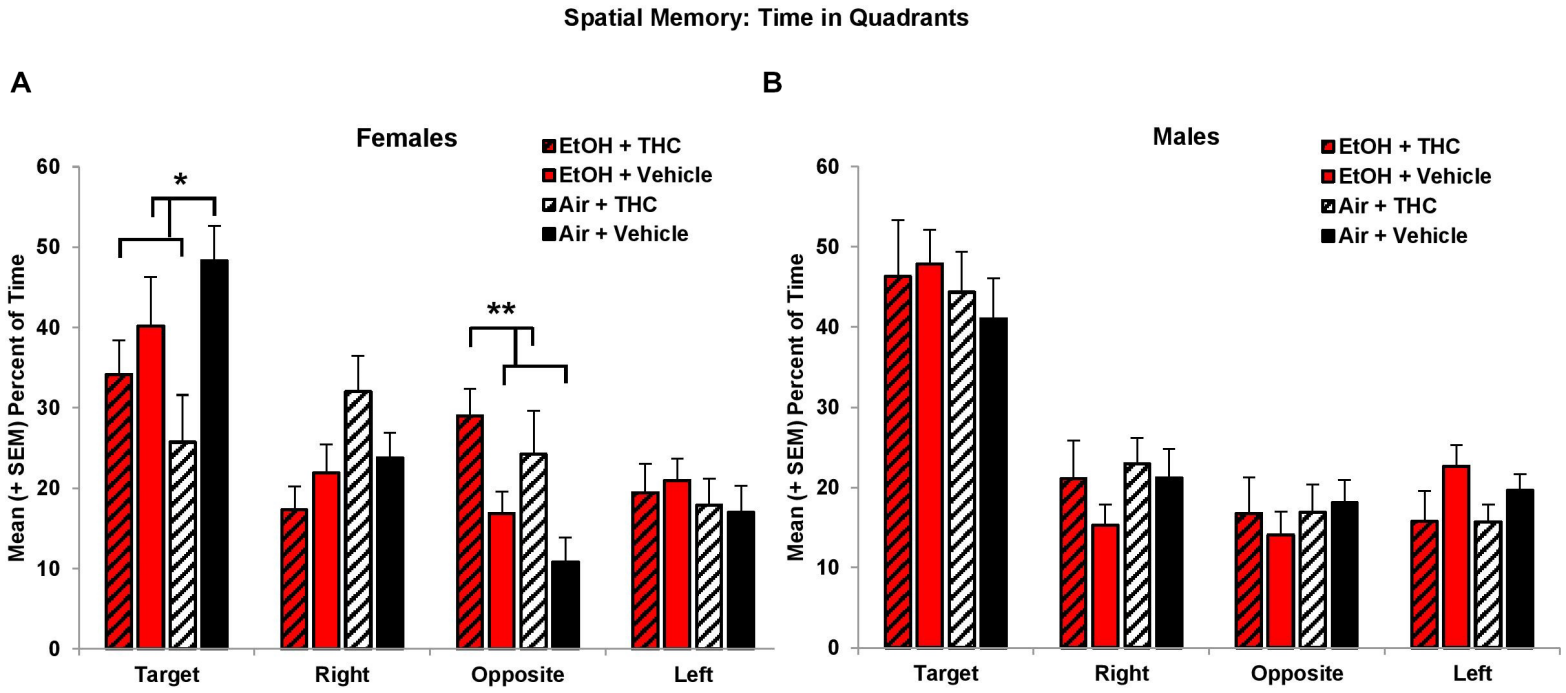
Female offspring exposed to THC spent less time in the Target quadrant and more time in the Opposite quadrant than non-exposed offspring **(A)**. Neither prenatal THC nor alcohol significantly affected spatial memory among male offspring **(B)**. *THC < no THC, *p* < 0.05; **THC > no THC, *p* < 0.01.

**Figure 6:**
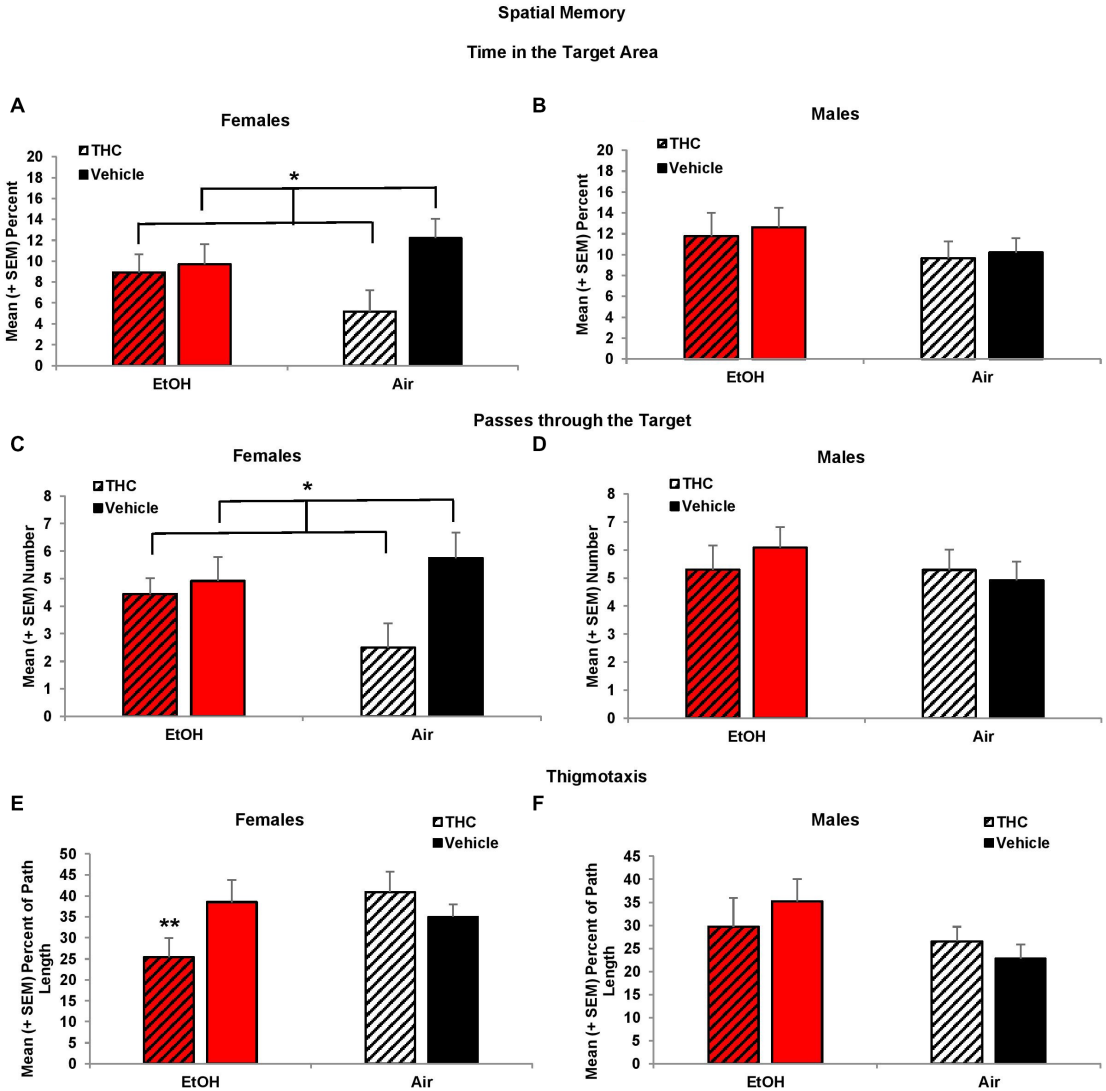
Female offspring exposed to prenatal THC showed impaired performance during the probe trial, spending less time in the target area **(A)** and fewer passes through the target **(C)**. Female offspring exposed to combined alcohol and THC prenatally were less thigmotaxic **(E)**. No differences were observed among male offspring **(B,D,F)**. *THC < no THC, *p* < 0.01; **EtOH + THC < EtOH + Vehicle and Air + THC, *p*’s < 0.05.

Consistent with acquisition deficits, females exposed to prenatal THC showed significant impairments on spatial memory during the probe trial. THC-exposed females spent less time in the platform quadrant (*F*[1,41] = 7.0, *p* < 0.01, Figure 5A) leading to a main effect of sex (*F*[1,85] = 4.2, *p* < 0.05) and interaction of Sex*THC (*F*[1,85] = 4.0, *p* < 0.05). Conversely, females exposed to prenatal THC increased time spent in the opposite quadrant (*F*[1,41] = 10.8, *p* < 0.01), which also led to an interaction of Sex*THC (*F*[1,85] = 5.4, *p* < 0.05) and a main effect of THC (*F*[1,85] = 6.7, *p* < 0.05). In fact, female subjects exposed to THC did not discriminate among the quadrants, spending roughly 25% time in each quadrant, as seen in Figure 5A. Females with prenatal THC exposure also spent less time (*F*[1,41] = 4.1, *p* < 0.05; Figure 6A) and made fewer passes (*F*[1,41] = 4.7, *p* < 0.05; Figure 6C) in the platform area.

Collapsed across sex, subjects exposed to EtOH alone exhibited greater thigmotaxis during the probe trial than the Combination (*p* < 0.05) group, resulting in a significant interaction between EtOH*THC (*F*[1,85] = 5.1, *p* < 0.05). However, analyses also revealed an EtOH*Sex interaction (*F*[1,85] = 4.9, *p* < 0.05) and a main effect of sex (*F*[1,85] = 4.2, *p* < 0.05). Upon separating the data by sex, it was found that females exposed to the combination of EtOH and THC spent less time in thigmotaxis than either drug exposure alone, resulting in an EtOH*THC interaction (*F*[1,41] = 4.4, *p* < 0.05; Figure 6E), a result that was consistent with acquisition (Figures 6B,D,F).

### 3.3. Working memory

During the working memory task, the location of the platform changes with each session, so during the training trials, subjects have no prior knowledge of the platform location. However, during the 0-s ITI training trials, there was a three-way interaction of Day*EtOH*THC (*F*[2,170] = 3.7, *p* < 0.05) and a main effect of Sex (*F*[1,85] = 8.7, *p* < 0.01), as females took longer path lengths than males. Females exposed to prenatal EtOH took shorter path lengths to find the platform compared to those not exposed to EtOH (EtOH + THC: *M* = 8.44 ± 0.82 m; EtOH: *M* = 8.44 ± 0.66; THC: *M* = 9.63 ± 0.60; Vehicle: *M* = 9.67 ± 0.41), although this effect failed to reach statistical significance (*F*[1,41] = 3.8, *p* = 0.06). Nevertheless, this suggests that females exposed to ethanol may utilize a different search strategy. There were no other effects of prenatal drug exposure or sex during training trials.

**Figure 7:**
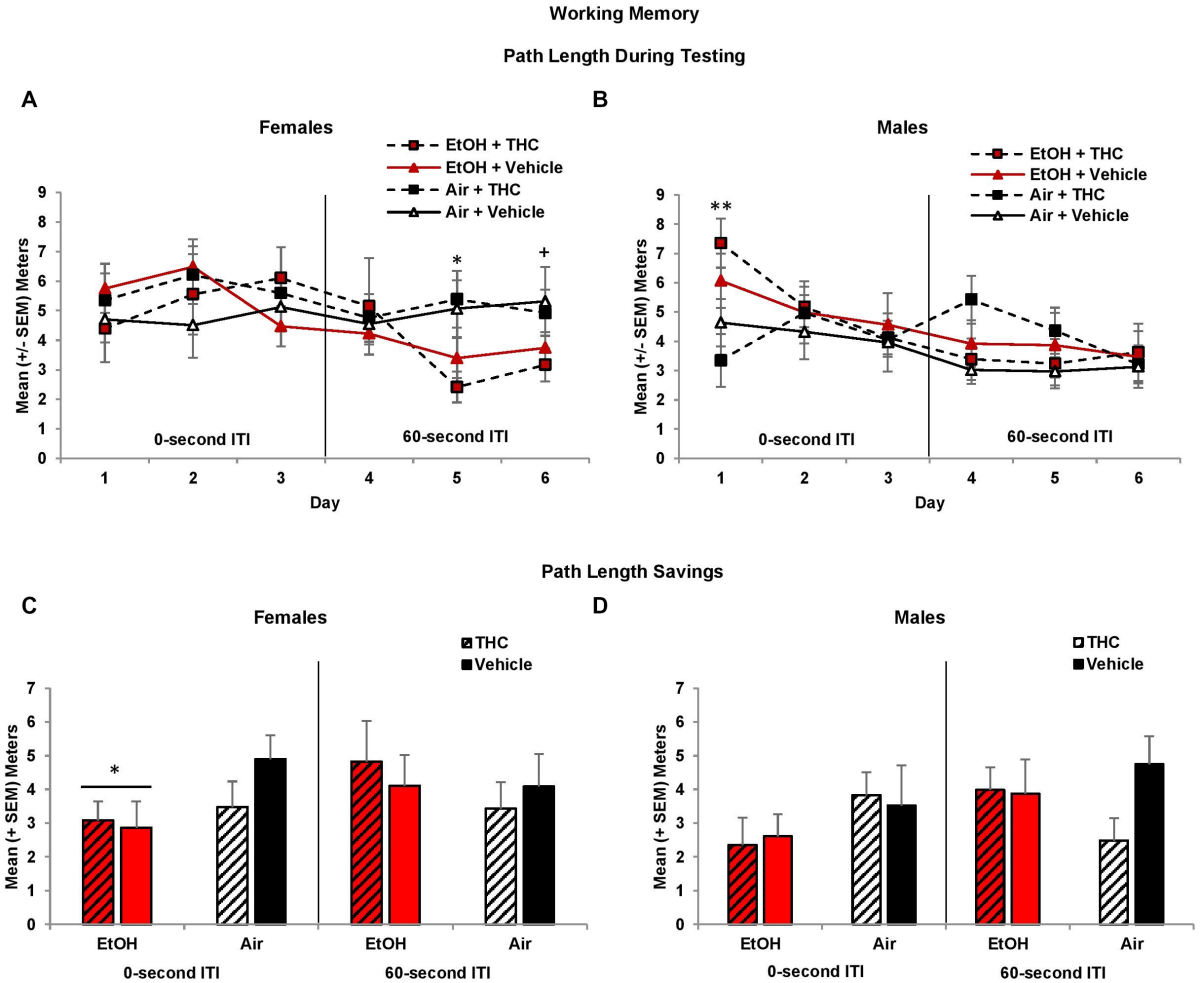
Male offspring exposed to prenatal alcohol exposure traveled longer path lengths on the first day of testing **(B)**, whereas female offspring with prenatal alcohol exposure traveled shorter path lengths during the latter 60-s ITI test trials **(A)**. However, offspring prenatally exposed to alcohol had less memory savings between the training and test trials, an effect driven by the female offspring **(C,D)**. *EtOH < no EtOH, *p*’s < 0.05; **EtOH > no EtOH, *p* < 0.05; ^+^EtOH < no EtOH, *p* = 0.06.

During the 0-s ITI testing sessions, an interaction of Day*Sex was observed (*F*[2,170] = 4.3, *p* < 0.05). Males prenatally exposed to EtOH required longer path lengths to find the platform on Day 1 (*F*[1,44] = 5.4, *p* < 0.05; Figure 7B). Consistent with memory impairments, males exposed to alcohol prenatally also had significantly larger heading angles during the 0-s ITI testing days (*F*[1,44] = 6.00, *p* < 0.05; EtOH + THC: *M* = 50.68 ± 4.73; EtOH: *M* = 47.37 ± 5.48; THC: *M* = 38.28 ± 3.21; Vehicle: *M* = 39.39 ± 2.95) producing an interaction of Sex*EtOH (*F*[1,85] = 5.0, *p* < 0.05). There were no effects among females (Figure 7A).

When the ITI was increased to 60-s, there was a main effect of Sex (*F*[1,85] = 4.2, *p* < 0.05), as well as a trending interaction of Day*EtOH*Sex (*F*[2,170] = 2.8, *p* = 0.06)Given the main effect of sex, data were analyzed separately for males and females. Among females, subjects exposed to prenatal EtOH took shorter path lengths to find the platform (*F*[1,41] = 5.7, *p* < 0.05; Figure 7A), particularly on Day 5 (*F*[1,41] = 9.3, *p* < 0.01) and 6 (*F*[1,41] = 3.5, *p* = 0.06). In contrast, female offspring exposed to THC had smaller heading angles on Day 5 only (*F*[1,41] = 4.1, *p* < 0.05; EtOH + THC: *M* = 34.91 ± 8.70; EtOH: *M* = 50.55 ± 7.19; THC: *M* = 45.00 ± 4.12; Vehicle: *M* = 61.80 ± 10.48). No significant differences were observed among male offspring (Figure 7B).

Memory performance is best measured as savings (testing minus training path lengths), which takes into account individual differences in training trial performance. Offspring exposed to prenatal EtOH exhibited less savings during the 0-s ITI sessions (*F*[1,85] = 4.8, *p* < 0.05). Although this effect was seen in both sexes, follow-up analyses indicated that EtOH impaired memory among female offspring (*F*[1,41] = 4.8, *p* < 0.05), but failed to reach statistical significance among males (Figures 7C,D). During the 60-s ITI sessions, despite a significant interaction of Day*Sex*EtOH (*F*[2,170] = 2.9, *p* = 0.05), follow-up analyses did not yield any meaningful or significant effects on individual Days (data not shown).

**Figure 8:**
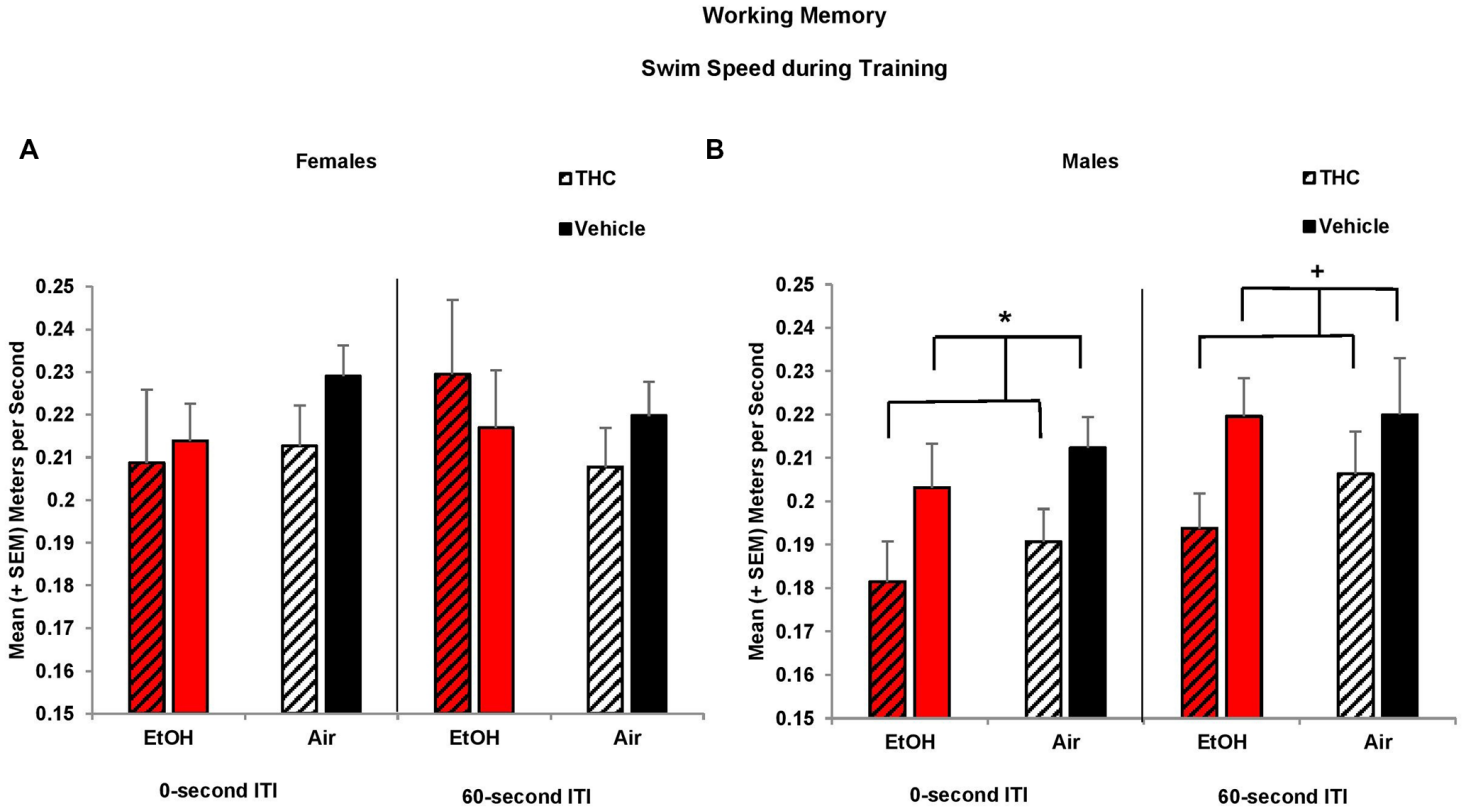
Male offspring exposed to prenatal THC swam slower during training sessions than those not exposed to THC **(B)**. Swim speed did not differ among groups of female offspring **(A)**. *THC < no THC, *p* < 0.05; ^+^THC < no THC, *p* = 0.06.

Similar to the Morris water maze spatial learning task, prenatal THC exposure reduced swimming speed among males during working memory training. Overall, all offspring increased their speed over Days (*F*[2,170] = 3.3, *p* < 0.05; data not shown), but offspring exposed to prenatal THC swam slower than those exposed to the Vehicle (*F*[1,85] = 5.8, *p* < 0.05). Given there was a significant effect of sex (*F*[1,85] = 6.3, *p* < 0.05), data were examined separately by sex. Reductions in speed among prenatal THC-exposed offspring during the 0-s training sessions were driven by the males (*F*[1,44] = 4.9, *p* < 0.05), not females (Figures 8A,B). A similar trend was also observed during the 60-s ITI training sessions among the male offspring (*F*[1,44] = 3.7, *p* = 0.06; Figures 8A,B). By the testing sessions, there were no meaningful group differences in swimming speed (data not shown). Thus, prenatal THC may influence motor function in males.

**Figure 9:**
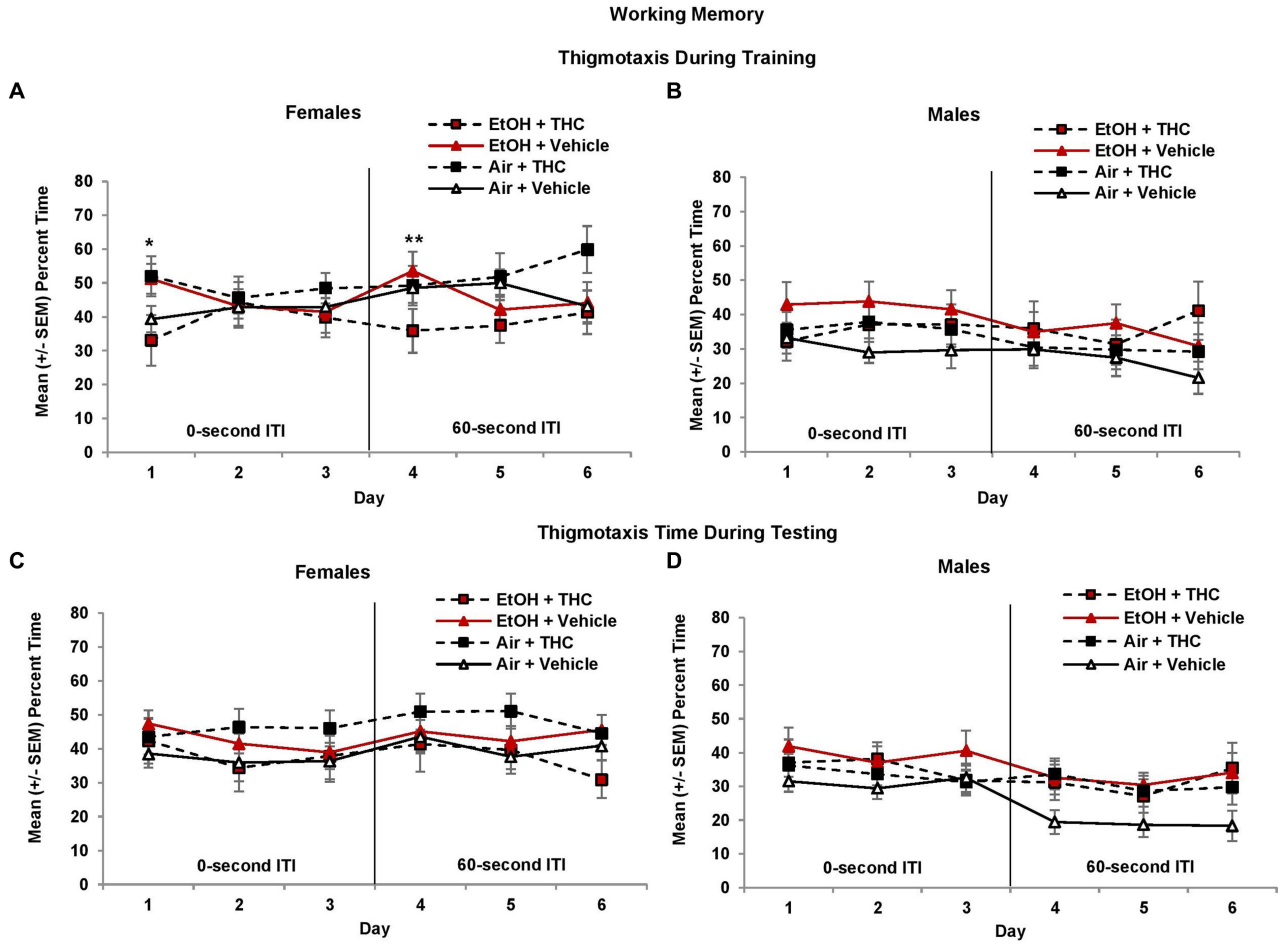
Female offspring exposed to either alcohol or THC prenatally spent more time in thigmotaxis on the 1st day of 0-s ITI training **(A)**. In contrast, females exposed to the combination of prenatal alcohol and THC spent less time in thigmotaxis on the 1st day of 60-s ITI training (4th day; **A**). There were no significant differences during testing in females **(C)**. No significant differences were observed among male offspring **(B,D)**. *EtOH + Vehicle and Air + THC > EtOH + THC and Air + Vehicle, *p* < 0.05; **EtOH + THC < all other groups, *p* = 0.05.

To determine if anxiety and/or alternative search strategies affected working memory performance, thigmotaxis was also examined. During the 0-s ITI training and testing sessions, female offspring spent more time in thigmotaxis than males (Training: *F*[1,85] = 4.1, *p* < 0.05; Testing: *F*[1,85] = 3.7, *p* = 0.05, Figures 9A,B). Among female offspring, an interaction of Day*EtOH*THC (*F*[2,82] = 4.8, *p* < 0.05) indicated females prenatally exposed to either EtOH (*F*[1,19] = 4.9, *p* < 0.05) alone or THC alone (*F*[1,22] = 4.1, *p* = 0.05) spent significantly more time in thigmotaxis than those exposed to the combination (EtOH + THC) or the Vehicle on the 1st day of 0-s ITI training (Figure 9A).

When the ITI was increased to 60 s, a Day*THC interaction was observed (*F*[2,170] = 7.5, *p* < 0.01) and a Sex*EtOH interaction approached significance (*F*[1,85] = 3.4, *p* = 0.06). Females exposed to the combination of prenatal EtOH + THC spent less time in thigmotaxis during the first 60-s ITI training session (Day 4) compared to those exposed to EtOH alone (*F*[1,19] = 4.2, *p* = 0.05, Figure 9A). During the 60-s ITI testing sessions, interactions of Day*Sex*THC (*F*[2,170] = 3.0, *p* = 0.05), and EtOH*THC (*F*[1,85] = 5.1, *p* < 0.05) were observed. Although female offspring exposed to prenatal THC generally spent more time in thigmotaxis, whereas males in the control group (Air + Vehicle) spent less time in thigmotaxis, effects were not significant (Figures 9C,D).

## 4. Discussion

The present study investigated the impact of combined prenatal exposure to EtOH and THC, delivered via an e-cigarette, on spatial and working memory. The results revealed sex-specific effects of THC and EtOH exposure on cognitive and emotional domains. Cognitively, prenatal THC exposure adversely impacted spatial learning and memory in female offspring, whereas prenatal EtOH exposure impaired working memory. Notably, the combination of prenatal exposure to THC and EtOH did not intensify the cognitive effects of either substance.

Surprisingly, our findings showed that ethanol exposure during gestation did not produce deficits in spatial learning and memory, in contrast to other preclinical studies using prenatal models (Patten et al., 2014; Brancato et al., 2020; Aglawe et al., 2021; Mahdinia et al., 2021) and 3rd trimester equivalent models (Thomas et al., 2007; Ryan et al., 2008; Breit et al., 2019b). Variation in outcome may be related to route of administration and/or developmental timing. However, our failure to find effects may be related to the lower peak BACs compared to previous studies. Specifically, spatial learning deficits are more often observed when peak BACs are over 200 mg/dl, whereas the peak BACs in the current study were around 150 mg/dl (albeit see below).

Clinical studies have shown that prenatal alcohol exposure can have dose-dependent negative impacts on learning, memory, and academic attainment in children and adults. For example, frequent episodes of binge drinking during the first and second trimester predict learning and memory deficits, including deficits in spatial memory, in children (Richardson et al., 2002). A recent study suggests that heavy, but not moderate, prenatal EtOH exposure impairs spatial learning and memory (Dodge et al., 2019). Importantly, in the Dodge et al. (2019) study, there was a confound of age, as the moderately exposed group was older, so there may be a developmental delay in performance that was missed in the moderately exposed group. Nevertheless, the amount of alcohol consumed per occasion was related to spatial navigation; thus, the absence of prenatal alcohol effect in the current study could be attributed to the failure to attain a threshold BAC.

In contrast, prenatal EtOH did impair spatial working memory. Females exposed to prenatal EtOH showed memory impairments during the 0-s ITI sessions, whereas males showed only transient impairments during the early stages of learning, as evidenced by longer path lengths and larger heading angles on the initial days of testing. Previous preclinical studies have also reported that prenatal alcohol exposure impairs spatial working memory (Schambra et al., 2017; Lucia et al., 2019; Waddell et al., 2020; Gursky et al., 2021), even at low to moderate ethanol doses (Schambra et al., 2017; Waddell et al., 2020) and across different gestational periods (Schambra et al., 2017; Lucia et al., 2019; Waddell et al., 2020; Gursky et al., 2021). Similar working memory deficits have been reported in clinical populations (Kodituwakku et al., 1995; Moore et al., 2021; Chetty-Mhlanga et al., 2022). This deficit is likely related to dysfunction of the prefrontal cortex (Waddell et al., 2020; Gursky et al., 2021), and our data suggest that the alcohol levels achieved in the current study may disrupt development of this brain area.

Unlike prenatal EtOH, our study found that exposure to prenatal THC did alter spatial learning and memory, but only in females. This sex effect contrasts with previous studies reporting that prenatal THC exposure impairs spatial memory in males (de Salas-Quiroga et al., 2020; Castelli et al., 2023), but not females (de Salas-Quiroga et al., 2020). Prenatal cannabinoid exposure has also been found to impact CB1 receptors and GABAergic transmission in the hippocampus of adult male rats, potentially affecting long-lasting learning and memory performance (Castelli et al., 2007; Beggiato et al., 2017; de Salas-Quiroga et al., 2020), whereas others report that prenatal THC exposure may not alter hippocampal function in females (de Salas-Quiroga et al., 2020). Interestingly, previous research within our own lab using a synthetic cannabinoid found no significant effects on spatial learning (Breit et al., 2019b). However, it is important to note that exposure in that study occurred during postnatal days 4–9, equivalent to the human third trimester, rather than prenatal exposure used in the current study. Importantly, differential effects of prenatal THC and EtOH on memory are consistent with individual and opposing effects each drug has on the hippocampus (Reid et al., 2021).

Despite deficits in spatial learning and memory, there was no effect of prenatal THC on working memory. Research on the effects of prenatal THC on working memory has yielded mixed findings in both preclinical (Silva et al., 2012; Beggiato et al., 2020; Castelli et al., 2023) and clinical studies (Smith et al., 2016; Murnan et al., 2021; Cioffredi et al., 2022). Studies have reported poorer performance on learning and memory tasks in children exposed to marijuana in the first trimester (Richardson et al., 2002) and an increased risk of educational underachievement for children of mothers with cannabis use disorder (Betts et al., 2022). However, adjusting for socioeconomic status and other comorbidities attenuated this association, suggesting that additional environmental factors may contribute to learning and memory outcomes. The inconsistencies in findings may be attributed to various factors such as the form of administration (injections, gavage), type of drug (THC, synthetic cannabinoids), dose, timing of exposure, and age of outcome measurement.

Unexpectedly, the combination of prenatal THC and EtOH did not produce more severe cognitive deficits than either drug alone. This was particularly surprising given pharmacokinetic interactions of prenatal EtOH and THC, which led to higher BACs (EtOH + THC = 200 mg/dl vs. EtOH = 150 mg/dl) and transiently elevated plasma THC levels (Breit et al., 2020). Moreover, both prenatal THC and alcohol exposure can disrupt endocannabinoid function, making offspring more vulnerable to stressors and impairing memory and learning processes (Trezza et al., 2012; Richardson et al., 2016; Little et al., 2021). The ECS plays a crucial role in various developmental processes, including movement, memory, and emotions; changes in its activity during periods of high brain plasticity, such as prenatal development, can lead to long-term problems in behavior and brain function (Pinky et al., 2019; Navarrete et al., 2020; Little et al., 2021; De Genna et al., 2022). For example, prenatal alcohol exposure is associated with increased EC activity and increased CB1 receptor activation (Nagre et al., 2015; Subbanna et al., 2015; Rodrigues et al., 2017), and prenatal alcohol exposure is associated with long-lasting changes in the ECS, which may contribute to some of the behavioral deficits observed in FASD (Basavarajappa, 2015; Hungund, 2017). In contrast, prenatal cannabinoid exposure actually reduces CB1 activation (de Salas-Quiroga et al., 2015, 2020). However, when consumed together, combined exposure increases the bioavailability of each drug (Abel and Subramanian, 1990; Hartman et al., 2015, 2016), which may lead to greater alterations in the ECS.

Both the hippocampus and prefrontal cortex are rich in CB1 receptors and, in fact, combined exposure to THC and alcohol can lead to more severe neuronal degeneration and altered neuronal circuitry in the hippocampus (Hansen et al., 2008; Basavarajappa, 2015). The lack of combination effects on cognitive functioning are also surprising given findings in other behavioral domains. For example, the combination of EtOH and CP-55,940 produces more severe motor deficits and hyperactivity (Breit et al., 2019a), as well thigmotaxis (Breit et al., 2019b). Even littermates from the present study exposed to the combination illustrated more severe open field hyperactivity compared to either substance alone (Breit et al., 2022). Thus, the combination of ethanol and THC at the doses used in the present study affects behavioral domains outside of cognitive functioning.

In fact, the combination EtOH and THC did reduce thigmotaxis among female subjects compared to either prenatal EtOH or THC alone, suggesting that the combination may impact emotional processing, reducing anxiety and increasing risk-taking behavior. Interestingly, zebrafish exposed to a low-dose CB1R agonist during development exhibit increased risk-taking behavior, but only when combined with alcohol (Boa-Amponsem et al., 2019). Moreover, littermates from the current study exposed to the combination of prenatal EtOH and THC were less thigmotaxic in the open field, spending more time in the center (Breit et al., 2022), although this effect was seen in males, but not females. Together, these data suggest that the combination of prenatal EtOH and THC may produce unique effects on emotional development.

In contrast to the combination, exposure to either prenatal EtOH or THC alone increased thigmotaxis, suggesting increased anxiety. For example, female subjects exposed prenatally to THC exhibited increased thigmotaxis during both spatial and working memory tasks. The effects of prenatal cannabis exposure on emotional development have been diverse and dependent on various factors, including the age of testing, cannabinoid type, and sex of the subjects. For instance, prenatal exposure to whole-plant cannabis extract increased anxiety-like behavior in juveniles but not in adults (Weimar et al., 2020), whereas studies of prenatal exposure to synthetic cannabinoids have reported mixed results, including no effects (Manduca et al., 2020), decreased anxiety/increased risk-taking (Boa-Amponsem et al., 2019; Breit et al., 2019b) and elevated anxiety-like behaviors (Traccis et al., 2021; Lallai et al., 2022). Interestingly, in littermates prenatally exposed to THC alone, there were no effects on open field thigmotaxis, although subjects were tested during pre-adolescence (Breit et al., 2022). Clinically, cannabis exposure has been associated with increased frequency of depression and anxiety in children (Goldschmidt et al., 2004; Leech et al., 2006; Eiden et al., 2018; Grant et al., 2020); thus, prenatal cannabis may impact emotional development in complex ways.

Similarly, prenatal EtOH exposure produced modest increases in thigmotaxis among females. As with prenatal THC, the effects of prenatal EtOH exposure on emotional development are highly variable, with some reporting increased anxiety (Balaszczuk et al., 2019; Aglawe et al., 2021), while others report decreased anxiety/more risk-taking behavior (Breit et al., 2019b), and with variable sex-dependent effects (Weinberg et al., 2008; Muñoz-Villegas et al., 2017; Bake et al., 2021) related to alterations in the hypothalamic–pituitary–adrenal axis (Weinberg et al., 2008). Clinical studies have also reported mixed results (Goldschmidt et al., 2004; O’Leary et al., 2010).

Interestingly, the effects of prenatal EtOH and THC on cognitive and emotional functioning in the present study were more robust among females. However, prenatal THC did reduce swimming speed among males. We previously reported that prenatal THC exposure can delay sensorimotor development and impair motor function in adolescents, although we did not see sex-specific effects on those outcomes (Breit et al., 2022). Thus, motor coordination deficits induced by prenatal THC exposure may persist in males, but not females, as they age. Although clinical research has found varied effects of prenatal THC on motor development, including no impairments (Chandler et al., 1996; Richardson et al., 2002; Huizink, 2014), impairments (Richardson et al., 1995), and advanced motor skills (Fried and Watkinson, 1990; Huizink, 2014), further investigation is required to discern the impact of cannabis on motor development and coordination, and to identify any possible sex differences.

Notably, in addition to behavioral effects, offspring prenatally exposed to THC exhibited lower weight compared to those not exposed to THC. These results illustrate that even low levels of prenatal THC exposure can impair growth and that growth impairments extend beyond early life (Breit et al., 2022) into adolescence. Importantly, this growth impairment was seen in both sexes. Given the sex- and task-dependent behavioral effects of prenatal THC, body weight reductions do not relate to behavioral outcome. Nevertheless, long-lasting effects of prenatal THC on physical development represent another adverse outcome associated with prenatal cannabis exposure.

The current study illustrates that prenatal exposure to EtOH and THC can alter cognitive and emotional development, but there are several limitations to consider. First, the effects of only one dose of THC and EtOH were examined, and consequences of either drug alone or in combination are likely dose-dependent. This is particularly critical to investigate, as the lack of regulation of THC products and the dramatic variation in THC potencies (Giroud et al., 2015; Kahr et al., 2015) create challenges in establishing dose–response effects in clinical studies. Second, only THC was administered, but cannabis products include many constituents which may impact outcome. By solely focusing on one constituent of cannabis, THC, the potential interactive effects of other cannabinoids present in various cannabis products, such as CBD, are not considered.

Third, the study focused primarily on learning and memory during late adolescence, without follow-up testing as they transitioned into adulthood. The age range was chosen because rats have typically reached the ontological stage of hippocampal maturation and spatial learning ability (Morterá and Herculano-Houzel, 2012), and are capable of navigating and swimming efficiently in the maze (D’Hooge and De Deyn, 2001; Albani et al., 2014). It is possible that performance could be affected by factors such as pubertal hormones. For instance, the onset of puberty has been associated with improved cognitive performance in the Morris Water Maze, possibly due to prefrontal cortex maturation and changes in learning strategy (Willing et al., 2016). Performance in females can also be affected by differences in the estrous cycle, while alterations in sex hormones can impact spatial ability in males (D’Hooge and De Deyn, 2001; Moradpour et al., 2013). However, recent data suggest that hormonal factors may not necessarily induce large changes in behavior (Levy et al., 2023). Importantly, we do not know if prenatal alcohol or THC is affecting pubertal onset in the current study; thus, future studies should examine whether alterations in maturation contribute to behavioral outcomes.

Fourth, there is a possibility that the subjects’ performance on the initial Morris water maze task could affect subsequent performance on the working memory version of the task. For example, if the memory for the initial spatial location of the escape platform is strong, subjects may become biased towards a particular strategy or location and have more difficulty adapting to the changing demands of the working memory task. Importantly, subjects were tested on the working memory version 1 week after spatial memory testing to reduce carryover effects and all subjects showed improvement in performance. Moreover, even if prior spatial learning impacted performance on the working memory task, impaired performance would still reflect deficits in cognitive flexibility. Nevertheless, testing subjects on only the working memory version would determine whether interference influenced performance.

Finally, the control subjects in his study were exposed to propylene glycol vehicle, a well-known e-cigarette vehicle commonly used in e-liquids alongside various flavorings. The effects of inhaling e-cigarette vehicles by themselves, as well as the effects of mixing various commonly used flavorings, remain poorly understood. In fact, a recent study discovered that e-cigarette vehicles can dysregulate gene expression in the lungs and that adding flavorings can increase toxicity and alter immune lung cells, potentially leading to reduced lung function (Szafran et al., 2020).

In summary, our findings suggest that prenatal exposure to THC and EtOH can have differential effects on cognitive functions, with sex-specific and substance-specific patterns. Our results suggest that even low doses of THC delivered through e-cigarettes during pregnancy can lead to learning and memory deficits, whereas exposure to EtOH can impair working memory. Co-administration of THC and EtOH did not magnify the adverse effects of either substance on cognitive function, but may influence anxiety-like behavior and risk-taking tendencies. Overall, our findings underscore the potential harm of THC and EtOH on fetal development and reinforce the importance of educating pregnant individuals and promoting public health policies and interventions to minimize the risk of drug-related cognitive deficits in offspring.

## Data availability statement

The raw data supporting the conclusions of this article will be made available by the authors, without undue reservation.

## Ethics statement

The animal study was reviewed and approved by Institutional Animal Care and Use Committee at San Diego State University.

## Author contributions

AL contributed as first authorship based on roles in study design, project organization, data collection, data analyses, and writing of the manuscript. KB contributed to study design, data collection, analyses, and writing of the manuscript. JT contributed as last authorship based on roles in study design, funding acquisition, supervision, data analyses, and writing of the manuscript. All authors contributed significantly to the preparation of this submission.

## Funding

This work was supported by NIAAA grants AA025425 and AA012446 to JT, the NIAAA training grant T32 AA007456-38 and the NIH Loan Repayment Program award to KB.

## Conflict of interest

The authors declare that the research was conducted in the absence of any commercial or financial relationships that could be construed as a potential conflict of interest.

## Publisher’s note

All claims expressed in this article are solely those of the authors and do not necessarily represent those of their affiliated organizations, or those of the publisher, the editors and the reviewers. Any product that may be evaluated in this article, or claim that may be made by its manufacturer, is not guaranteed or endorsed by the publisher.
